# Supplementary material for: Polymorphism of feldspars above 10 GPa
Source: Nat Commun. 2020 Jun 1;11:2721. doi: 10.1038/s41467-020-16547-4 (PMC7264230; doi:10.1038/s41467-020-16547-4)
Supplement: Supplementary file 1 — Supplementary Information [file 41467_2020_16547_MOESM1_ESM.pdf]

Supplementary Information

for the manuscript “Polymorphism of feldspars above 10 GPa”

by Anna Pakhomova et al.

Supplementary Figure 1. Pressure-induced evolution of unit-cell parameters of previously known phases of anorthite (red), albite (green) and microcline (blue). Our data are presented with filled markers while previously published data are given as open markers. The *c* axis for anorthite is twice reduced to enable comparison with other feldspars. The errors are smaller than the markers sizes.

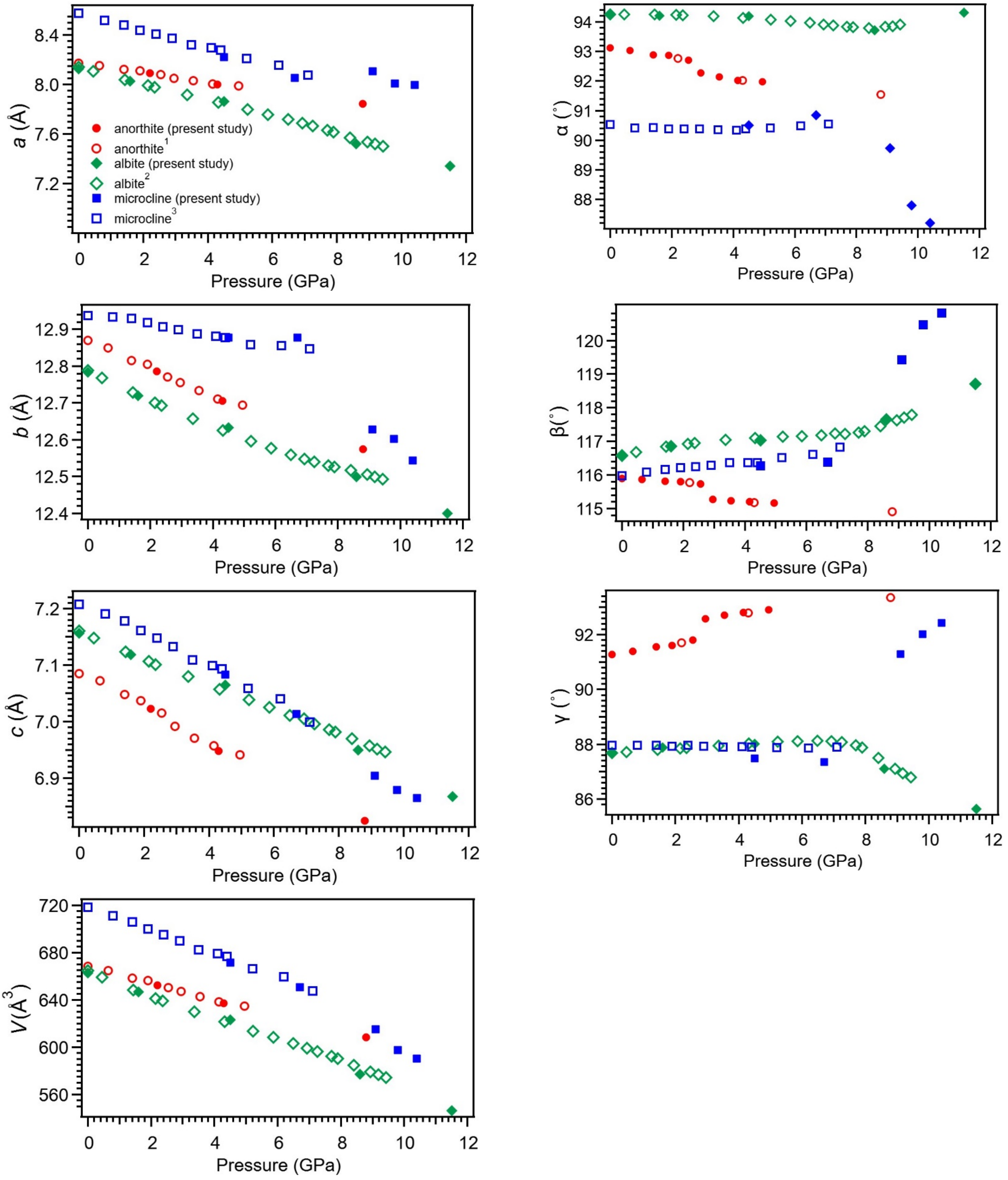

Supplementary Figure 2. Mechanism of Al coordination number increase upon compression of microcline. a) Fragment of the crystal structure of microcline at ambient conditions showing progressive closure of six-membered rinds and continuous approach of additional O3 atom into Al coordination sphere. The shortening Al-O3 contact is shown as a dashed grey line. b) Pressure-dependence of bond angle variance (BAV,˚) of AlO_4 tetrahedra. c) The evolution of quadratic elongation (QE) of AlO_4 tetrahedra along the compression. The parameters QE and BAV that show deviation of AlO_4 polyhedra from geometry of ideal tetrahedron are defined as[4]:

QE = $\frac{1}{4}\sum_{i=1}^{4}\left(\frac{l_i}{l_0}\right)^2$ and BAV = $\sqrt{\frac{1}{5}\sum_{i=1}^{6}(\theta_i-109.47)^2}$ where l_0 is a center-to-vertex distance for ideal tetrahedron whose volume is equal to that of the distorted tetrahedron with bond lengths l_i and bond angles θ_i.

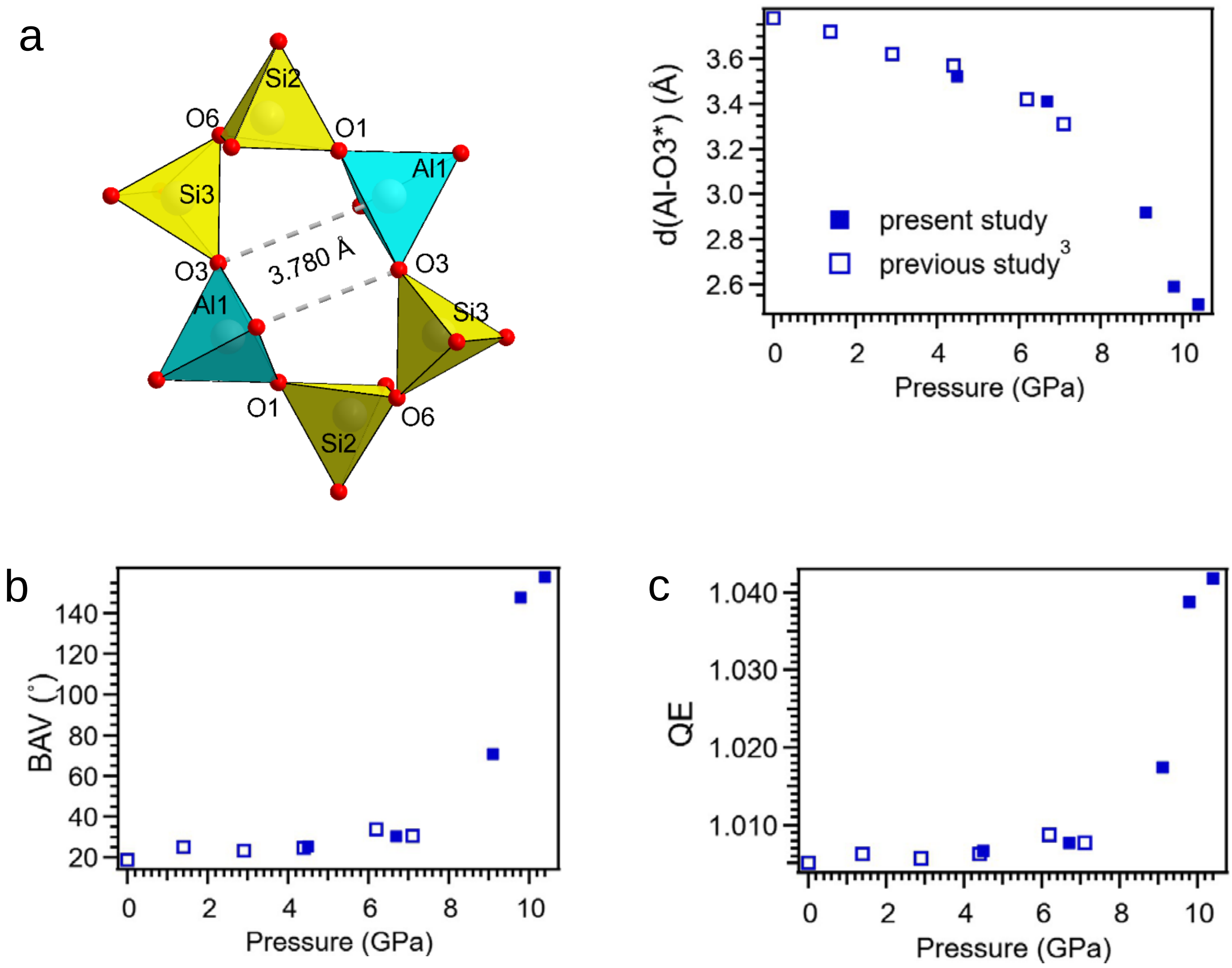

Supplementary Figure 3. Design of the BX110 diamond anvil cell

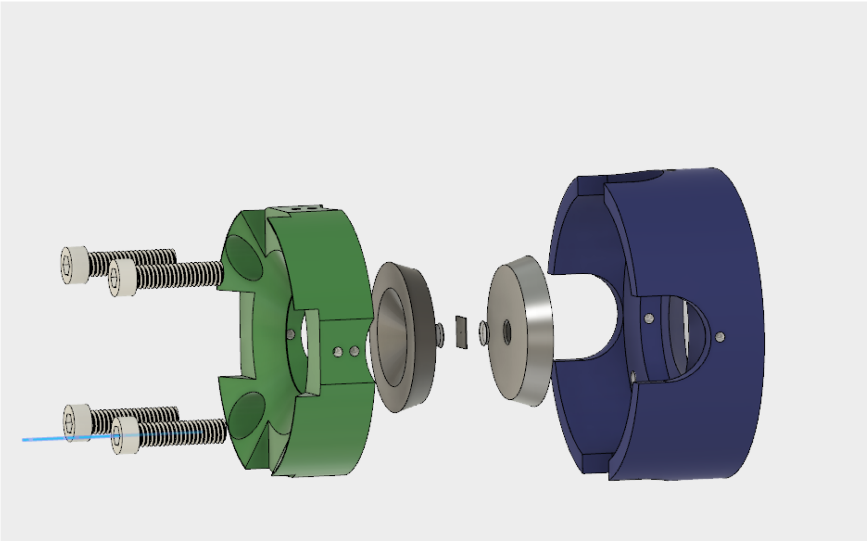

Supplementary Figure 4. Design of a Boehler-Almax diamond used in BX110

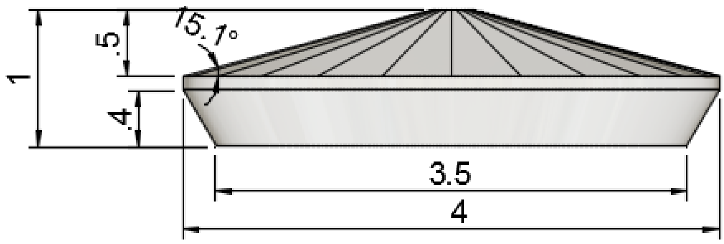

Supplementary Table 1. Evolution of unit-cell parameters, volume per number of formula units and density along the compression of anorthite, albite and microcline.

**№**	**Pressure, GPa**	**Sp.Gr.**	***a*, Å**	***b*, °**	***c*, Å**	***α*, °**	***β*, °**	***γ*, °**	***V*, Å^3**	***V/Z*, Å^3**	**density, g/cm^3**
Anorthite											
p01	2.2(1)	*P*-1	8.0922(6)	12.7858(9)	14.045(3)	92.765(12)	115.775(14)	91.705(6)	1305.0(4)	163.12(5)	2.832
p02	4.3(1)	*I*-1	7.9989(6)	12.706(2)	13.896(2)	92.020(14)	115.180(9)	92.788(10)	1274.1(4)	159.26(5)	2.901
p03	8.8(1)	*I*-1	7.8447(6)	12.574(2)	13.649(2)	91.549(14)	114.911(9)	93.347(10)	1216.9(3)	152.11(4)	3.037
p04	11.1(1)	*P*-1	7.6454(8)	11.944(1)	12.906(3)	96.677(13)	109.590(14)	80.210(8)	1091.9(3)	136.49(4)	3.386
p05	16.2(1)	*P*-1	7.5956(10)	11.8527(13)	12.829(4)	96.913(16)	109.335(17)	80.036(10)	1070.9(4)	133.86(5)	3.451
p06	16.4(1)	*P*-1	7.5954(11)	11.8628(11)	12.803(3)	96.907(14)	109.383(17)	80.003(9)	1069.2(4)	133.65(5)	3.457
Albite											
p01	1.6(1)	*C*-1	8.028(3)	12.720(3)	7.1188(18)	94.207(17)	116.85(3)	87.88(2)	646.8(3)	161.70(8)	2.693
p02	8.6(1)	*C*-1	7.519(2)	12.499(2)	6.9499(14)	93.723(14)	117.66(3)	87.109(19)	577.1(3)	144.28(8)	3.018
p03	11.5(1)	*C*-1	7.343(3)	12.400(3)	6.8671(9)	94.311(13)	118.71(3)	85.64(3)	546.3(3)	136.55(5)	3.189
p04	16.0(1)	*P*-1	6.984(2)	6.911(2)	11.735(3)	114.21(2)	87.57(3)	108.31(3)	488.0(3)	122.00(8)	3.569
p05	17.5(1)	*P*-1	6.9825(8)	6.9058(5)	11.7294(12)	113.650(7)	92.688(10)	71.088(9)	487.86(9)	121.96(2)	3.570
p06	20.5(1)	*P*-1	6.9489(7)	6.8529(3)	11.5562(10)	113.094(5)	92.427(8)	71.249(6)	477.12(7)	119.28(2)	3.651
p07	13.5(1)	*P*-1	6.585(2)	6.8852(9)	7.0218(9)	63.648(15)	64.15(3)	75.641(19)	256.13(12)	128.06(6)	3.400
p08	4.5(1)	*C*-1	7.8619(8)	12.6336(7)	7.0643(10)	94.185(7)	117.034(12)	88.013(5)	623.32(13)	155.83(2)	2.794
p09	0.00001	*C*-1	8.1238(7)	12.7836(6)	7.1566(12)	94.274(7)	116.570(13)	87.692(5)	662.86(15)	165.63(2)	2.628
Microcline											
p01	4.5(1)	*C*-1	8.2204(4)	12.8776(4)	7.0828(3)	90.502(2)	116.271(4)	87.488(6)	671.64(5)	167.91(1)	2.753
p02	6.7(1)	*C*-1	8.0517(11)	12.8772(11)	7.0137(7)	90.849(7)	116.381(12)	87.361(14)	650.77(14)	162.69(4)	2.841
p03	9.1(1)	*C*-1	8.1046(12)	12.6276(11)	6.9043(7)	89.738(7)	119.429(14)	91.297(9)	615.25(14)	153.81(4)	3.005
p04	9.8(1)	*C*-1	8.006(2)	12.602(3)	6.8794(8)	87.802(11)	120.47(2)	92.01(2)	597.65(18)	149.41(4)	3.094
p05	10.4(1)	*C*-1	7.996(3)	12.544(4)	6.8650(12)	87.204(16)	120.82(3)	92.42(3)	590.5(3)	147.62(6)	3.131
p06	12.8(1)	*P*-1	6.3077(11)	6.7648(13)	7.0291(13)	74.068(17)	85.016(14)	64.844(18)	260.91(9)	130.46(4)	3.543
p07	13.9(1)	*P*-1	6.2933(15)	6.7430(19)	7.006(2)	74.26(3)	85.38(2)	64.85(3)	258.80(14)	129.40(7)	3.572
p08	14.9(1)	*P*-1	6.2737(12)	6.7304(15)	6.9896(16)	74.14(2)	85.035(18)	64.86(2)	256.87(11)	128.44(6)	3.598
p09	16.1(1)	*P*-1	6.2497(11)	6.7162(14)	6.9953(13)	74.011(17)	85.108(15)	64.899(18)	255.44(9)	127.72(4)	3.619
p10	17.8(1)	*P*-1	6.2233(8)	6.6957(10)	6.9787(10)	74.120(13)	85.229(11)	64.844(13)	252.99(7)	126.50(4)	3.654
p11	19.6(1)	*P*-1	6.1770(12)	6.6728(16)	6.9367(16)	74.36(2)	85.072(17)	64.62(2)	248.59(11)	124.30(4)	3.719
p12	23.0(1)	*P*-1	6.1219(13)	6.6625(15)	6.9178(14)	74.371(19)	85.418(17)	64.25(2)	244.51(10)	122.52(5)	3.781
p13	24.8(1)	*P*-1	6.0910(11)	6.6445(15)	6.9011(17)	74.23(2)	85.363(17)	64.07(2)	241.49(10)	120.74(5)	3.828
p14	26.8(1)	*P*-1	6.0538(12)	6.6411(16)	6.9101(17)	73.99(2)	85.729(18)	63.90(2)	239.45(10)	119.72(5)	3.861

Supplementary Table 2. Crystallographic data and refinement parameters for the new high pressure phases of anorthite (An), albite (Ab) and microcline (Mc)

Crystal data	An-III	Ab-II	Ab-III	Mc-II
Pressure, GPa	11.1(1)	13.5(1)	17.5(1)	12.8(1)
Space group	*P*-1	*P*-1	*P*-1	*P*-1
a, Å	7.6454(8)	6.585(2)	6.9825(8)	6.3077(11)
b, Å	11.9437(11)	6.8852(9)	6.9058(5)	6.7648(13)
c, Å	12.906(3)	7.0218(9)	11.7294(12)	7.0291(13)
α, °	96.677(13)	63.648(15)	113.650(7)	74.068(17)
β, °	109.590(14)	64.15(3)	92.688(10)	85.016(14)
γ, °	80.210(8)	75.641(19)	71.088(9)	64.844(18)
Volume, $Å^3$	1091.9(3)	256.13(12)	487.86(9)	260.91(9)
Z	8	2	4	2
Data collection				
Wavelength	0.2889	0.2894	0.2952	0.2907
Max. θ°	11.909	15.244	14.949	12.92
Index ranges	$-10 \leq h \leq 10$	$-8 \leq h \leq 4$	$-9 \leq h \leq 7$	$-9 \leq h \leq 9$
	$-17 \leq k \leq 16$	$-12 \leq k \leq 12$	$-12 \leq k \leq 12$	$-10 \leq k \leq 10$
	$-11 \leq l \leq 8$	$-11 \leq l \leq 10$	$-18 \leq l \leq 16$	$-10 \leq l \leq 9$
No.meas.refl.	2488	963	1760	1573
No.uniq.refl.	1587	740	1160	1026
No. obs.refl ($I > 2\sigma(I)$)	1297	647	1088	866
Refinement of the structure				
No.of variables	229	78	156	118
R_{int}	0.0449	0.0245	0.0138	0.0249
R_1, all data	0.0773	0.0841	0.0418	0.0641
R_1, $I > 2\sigma(I)$	0.0874	0.0798	0.0433	0.0551
wR_2, all data	0.2085	0.2333	0.1140	0.1679
wR_2, $I > 2\sigma(I)$	0.2294	0.2257	0.1164	0.1530
GooF	1.097	1.136	1.073	1.083

Supplementry Table 3. Bond distances in anorthite-III, $CaAl_2Si_2O_8$, at 11.1 GPa

**Si1**	O26	1.550(16)	**Si2**	O32	1.57(3)	**Si3**	O20	1.572(8)	**Si4**	O19	1.57(3)
	O5	1.579(8)		O6	1.579(8)		O4	1.60(2)		O16	1.587(8)
	O2	1.591(9)		O9	1.59(1)		O31	1.64(4)		O27	1.62(2)
	O33	1.68(3)		O30	1.61(2)		O28	1.671(9)		O10	1.62(1)
	av.	1.599		av.	1.585		av.	1.621		av.	1.600
**Si5**	O24	1.566(12)	**Si6**	O8	1.55(5)	**Si7**	O11	1.54(2)	**Si8**	O23	1.57(2)
	O15	1.60(2)		O21	1.603(9)		O12	1.57(2)		O7	1.58(1)
	O17	1.630(9)		O3	1.61(3)		O13	1.61(1)		O14	1.61(2)
	O25	1.65(2)		O18	1.62(1)		O22	1.65(1)		O1	1.65(2)
	av.	1.610		*av.*	1.597		*av.*	1.594		*av.*	1.604
**Al1**	O2	1.71(2)	**Al2**	O33	1.64(3)	**Al3**	O27	1.66(3)			
	O10	1.725(9)		O7	1.754(9)		O16	1.714(8)			
	O24	1.768(8)		O22	1.763(7)		O5	1.744(13)			
	O26	1.83(3)		O1	1.765(18)		O28	1.76(2)			
	av.	1.757		*av.*	1.730		*av.*	1.718			
**Al4**	O12	1.727(12)	**Al5**	O31	1.726(16)	**Al6**	O4	1.752(10)			
	O11	1.749(8)		O20	1.766(8)		O15	1.77(2)			
	O23	1.783(9)		O17	1.82(3)		O32	1.80(2)			
	O19	1.78(3)		O3	1.83(3)		O17	1.85(2)			
	O7	2.22(3)		O25	1.908(11)		O25	1.949(7)			
	av.	1.853		*av.*	1.811		*av.*	1.824			
**Al7**	O6	1.740(19)	**Al8**	O18	1.77(3)						
	O21	1.745(19)		O8	1.85(2)						
	O9	1.77(2)		O30	1.872(17)						
	O14	1.907(14)		O30	1.876(7)						
	O13	2.156(17)		O13	1.919(8)						
	O1	2.16(2)		O22	2.283(16)						
	av.	1.916		*av.*	1.928						
**Ca1**	O4	2.301(14)	**Ca2**	O32	2.222(16)	**Ca3**	O8	2.142(17)	**Ca4**	O24	2.289(19)
	O9	2.34(2)		O28	2.259(9)		O14	2.229(12)		O3	2.326(7)
	O13	2.437(8)		O20	2.325(9)		O6	2.372(8)		O21	2.382(18)
	O11	2.44(2)		O33	2.392(10)		O19	2.391(7)		O19	2.395(11)
	O28	2.46(2)		O15	2.395(18)		O3	2.403(14)		O2	2.455(7)
	O33	2.532(10)		O26	2.45(2)		O10	2.41(2)		O23	2.49(3)
	O1	2.60(2)		O22	2.46(2)		O25	2.48(2)		O27	2.50(2)
	O5	2.64(2)		O18	2.95(2)		O12	2.62(2)		O14	2.697(18)
	O32	2.729(6)								O31	2.99(3)
	av.	2.496		*av.*	2.432		*av.*	2.381		*av.*	2.503

Supplementary Table 4. Bond distances in albite-II, $NaAlSi_3O_8$, at 13.5 GPa

**Si1**	O6	1.562(11)	**Si2**	O2	1.578(11)	**Si3**	O3	1.572(9)
	O4	1.591(4)		O7	1.596(5)		O2	1.604(4)
	O7	1.633(5)		O1	1.614(4)		O8	1.608(10)
	O5	1.665(8)		O1	1.626(8)		O5	1.616(3)
	av.	1.613		*av.*	1.604		*av.*	1.600
**Al1**	O6	1.789(7)	**Na1**	O7	2.166(5)			
	O3	1.802(7)		O3	2.170(5)			
	O4	1.875(8)		O6	2.214(7)			
	O1	1.880(9)		O2	2.392(10)			
	O1	1.903(4)		O3	2.451(7)			
	O5	2.168(5)		O4	2.568(6)			
	av.	1.850		O1	2.621(7)			
				O8	2.713(10)			
				av.	2.412			

Supplementary Table 5. Bond distances in albite-III, $NaAlSi_3O_8$, at 17.5 GPa

**Si1**	O4	1.552(6)	**Si2**	O16	1.561(5)	**Si3**	O8	1.597(2)
	O11	1.583(2)		O12	1.586(3)		O13	1.598(5)
	O8	1.606(3)		O3	1.601(4)		O14	1.605(5)
	O6	1.664(4)		O10	1.656(3)		O10	1.627(2)
	av.	1.601		*av.*	1.601		*av.*	1.607
**Si4**	O15	1.581(2)	**Si5**	O5	1.579(5)	**Si6**	O12	1.653(4)
	O7	1.591(5)		O3	1.598(5)		O2	1.684(2)
	O13	1.604(5)		O15	1.603(2)		O1	1.718(4)
	O6	1.623(2)		O2	1.626(2)		O9	1.722(5)
	av.	1.600		*av.*	1.601		O9	1.778(2)
							O10	2.310(4)
							av.	1.711/1.811
**Al1**	O5	1.726(2)	**Al2**	O16	1.766(4)			
	O7	1.743(3)		O4	1.805(4)			
	O1	1.799(3)		O14	1.834(3)			
	O9	1.849(5)		O11	1.839(4)			
	O1	1.924(6)		O14	1.946(4)			
	av.	1.808		O6	1.966(2)			
				av.	1.859			
**Na1**	O7	2.151(3)	**Na2**	O2	2.138(4)			
	O8	2.160(3)		O16	2.170(4)			
	O16	2.226(3)		O4	2.173(4)			
	O11	2.314(3)		O7	2.341(4)			
	O3	2.390(5)		O5	2.400(6)			
	O2	2.680(6)		O13	2.507(4)			
	O14	2.733(5)		O1	2.546(2)			
	av.	2.379		av.	2.325			

Supplementary Table 6. Bond distances in microcline-II, $KAlSi_3O_8$, at 12.8 GPa

**Si1**	O1	1.552(3)	**Si2**	O8	1.560(4)	**Si3**	O6	1.601(3)
	O7	1.582(4)		O4	1.612(6)		O3	1.606(6)
	O5	1.635(4)		O6	1.649(3)		O5	1.613(4)
	O2	1.669(6)		O2	1.651(5)		O4	1.635(5)
	av.	1.610		*av.*	1.618		*av.*	1.614
**Al1**	O1	1.763(3)	**K1**	O4	2.521(4)			
	O7	1.800(4)		O8	2.572(3)			
	O8	1.805(6)		O7	2.620(6)			
	O3	1.882(5)		O1	2.648(6)			
	O3	1.918(4)		O5	2.667(4)			
	O2	2.163(3)		O6	2.670(3)			
	av.	1.889		O5	2.685(5)			
				O3	2.711(4)			
				O5	2.869(5)			
				av.	2.663			

Supplementary Table 7. Details of the multi anvil experiments

Starting material	Pressure (GPa)	Temperature (ºC)	Time (hour)	Recovered samples	Texture of the quenched sample
albite	12	800	2	jadeite+stishovite	polycrystalline
albite	13	800	2	jadeite+stishovite	polycrystalline
albite	17	700	2	jadeite+stishovite	polycrystalline
anorthite	13	300	2	anorthite	single crystals
anorthite	13	500	2	anorthite	single crystals
anorthite	13	700	2	pyroxene	single crystals
anorthite	14	600	2	anorthite	single crystals
anorthite	15	600	2	anorthite	single crystals
anorthite	15	1500	2	pyroxene	single crystals
anorthite	15	800	2	pyroxene+grossular	polycrystalline

Supplementary Table 8. Details of the crystal structure refinement of Ca-Al pyroxene (quenched from 15 GPa and 700 ºC)

Formula	$(Al_{0.47(1)}Ca_{0.53(1)})Ca(Si_{1.6}Al_{0.4})O_6$
Space group	*C*2/*c*
a, Å	9.7405(6)
b, Å	8.8710(5)
c, Å	5.3050(3)
β, °	105.910(6)
Volume, Å^3	440.83(5)
Z	4
Data collection	
Wavelength	0.56087
Max. θ°	28.590
Index ranges	$-10 \le h \le 16$
	$-15 \le k \le 10$
	$-8 \le l \le 7$
No.meas.refl.	2693
No.uniq.refl.	1045
No. obs.refl ($I > 2\sigma(I)$)	1011
No.of variables	48
R_{int}	0.0188
R_1, all data	0.0297
R_1, $I > 2\sigma(I)$	0.0290
wR_2, all data	0.0825
wR_2, $I > 2\sigma(I)$	0.0819
GooF	1.148

Supplementary Table 9. The feldspar compositions in wt% .

	Anorthite (An)	Albite (Ab)	Microcline (Mi)
**Na_2O**	0.18(0.02)	11.28(0.31)	0.67(0.12)
**SiO_2**	42.18(0.14)	65.14(0.99)	63.24(0.11)
**K_2O**	0.05(0.01)	0.24(0.13)	15.40(0.21)
**FeO**	0.12(0.02)	0.05(0.03)	0.02(0.02)
**Al_2O_3**	36.21(0.08)	20.74(0.49)	18.40(0.11)
**MgO**	0.007(0.006)	0.003(0.003)	0.003(0.003)
**CaO**	19.60(0.07)	1.00(0.39)	0.01(0.01)
**Sum**	**98.35**	**98.46**	**97.75**

Supplementary Table 10. Details of the *in situ* high-pressure single crystal diffraction experiments.

	#1	#2	#3	#4
**Sample**	Anorthite (An)	Albite (Ab)		Microcline (Mi)
**Composition**	$Ca_{1.00}Na_{0.02}Si_{1.99}Al_{2.01}O_8$	$Na_{0.98}Ca_{0.04}K_{0.01}Si_{2.91}Al_{1.09}O_8$		$K_{0.93}Na_{0.06}Si_{2.98}Al_{1.02}O_8$
**DAC type / culet size (μm)**	symmetric Mao-type / 300	BX110 / 250		BX110 / 250
**Pressure standard**	ruby sphere	gold foil		gold foil
**PTM***	Ne	Ne		Ne
**Studied pressure range (GPa)**	2-22	1-20	20-ambient pressure, decompression	1-27
**Experimental station**	DESY, P02.2 beamline	APS, 13-IDD beamline	DESY, P02.2 beamline	DESY, P02.2 beamline
**Wavelength (Å)**	0.2889	0.2952	0.2894	0.2907
**X-ray focusing / size (μm^2)**	KB mirrors / 2x2	KB mirrors / 3x3	KB mirrors / 2x2	KB mirrors / 2x2
**Detector**	Perkin Elmer 621	Pilatus CdTe 1M	Perkin Elmer 1621	Perkin Elmer 1621

*PTM = pressure transmitting media

Supplementary Note 1.

Design of BX110 diamond anvil cell

The design of BX110 diamond anvil cell is similar to preceding BX90[5] but features even larger conical opening of 110° (Supplementary Figure 3). In order to support such wide opening angle, special type of Boehler-Almax (BA) diamonds and seats are employed. While 90° BA diamonds are of 1.9 mm in height, the anvils with optical opening of 120° are shortened to 1mm and the cut is modified (Supplementary Figure 4) to decrease height to width ratio of culet-seat system. Particularly, height of the crown of 120° anvils is decreased to 0.42 mm resulting in the reduction of seat-anvil contact area to ~5.7 mm^2 which is ~1.5 times smaller in comparison to 90° BA diamonds. The operation with the BX110 is nearly identical to BX90. Seats with glued anvils are placed in the grooves of piston and cylinder. Four small screws allow precise regulation of seats position in the plane orthogonal to the load direction. Pressure between opposing diamonds is produced by applying loading force to the piston and cylinder, either with gas-driven membranes or with rotation of M4 screws.

Supplementary Note 2.

Transformation matrices used for conversion from the reduced unit cells into the reported unit cells.

Anorthite-III 1 0 0 0 -1 0 -1 0 -1

Albite-II 0 -1 0 -1 0 0 1 0 -1

Supplementary References

1. Angel, R. J., Hazen, R. M., McCormick, T. C., Prewitt, C. T. & Smyth, J. R. Comparative compressibility of end-member feldspars. Phys. Chem. Miner. 15, 313–318 (1988).

2. Benusa, M. D., Angel, R. J. & Ross, N. L. Compression of albite, $NaAlSi_3O_8$. Am. Mineral. 90, 1115–1120 (2015).

3. Allan, D. R. & Angel, R. J. A high-pressure structural study of microcline ($KAlSi_3O_8$) to 7 GPa. Eur. J. Mineral. 9, 263–276 (1997).

4. Robinson, K., Gibbs, G. V. & Ribbe, P. H. Quadratic Elongation: A Quantitative Measure of Distortion in Coordination Polyhedra. Science 172, 567–570 (1971).

5. Kantor, I. et al. BX90: A new diamond anvil cell design for X-ray diffraction and optical measurements. Rev. Sci. Instrum. 83, 125102 (2012).
