# Supplementary material for: Polymorphism of feldspars above 10 GPa
Source: Nat Commun. 2020 Jun 1;11:2721. doi: 10.1038/s41467-020-16547-4 (PMC7264230; doi:10.1038/s41467-020-16547-4)
Supplement: Supplementary file 3 — Description of Additional Supplementary Files [file 41467_2020_16547_MOESM3_ESM.pdf]

Description of Additional Supplementary Files

File Name: Supplementary Data 1
Description: Crystallographic Information Files for feldspars anorthite, albite and microcline studied at high pressures.
